# Lignin nanoparticle-enhanced PVA foils for UVB/UVC protection

**DOI:** 10.1038/s41598-025-19753-6

**Published:** 2025-10-13

**Authors:** Marta Goliszek-Chabros, Nataliia Smyk, Taroan Xu, Arkadiusz Matwijczuk, Beata Podkościelna, Olena Sevastyanova

**Affiliations:** 1 Analytical Laboratory, Institute of Chemical Science, Faculty of Chemistry, Maria Curie-Skłodowska University, M. Curie-Skłodowska Sq. 3, Lublin, 20-031 Poland; 2 Department of Analytical Chemistry, Faculty of Chemistry, Taras Shevchenko National University of Kyiv, Hetman Pavlo Skoropadsky 12, Kyiv, 01033 Ukraine; 3 Wallenberg Wood Science Center, Department of Fiber and Polymer Technology, School of Chemistry, Biotechnology and Health, KTH Royal Institute of Technology, Teknikringen 56-58, Stockholm, 100 44 Sweden; 4 Department of Biophysics, Institute of Molecular Biophysics, Faculty of Environmental Biology, University of Life Sciences in Lublin, Akademicka 13, Lublin, 20-950 Poland; 5 ECOTECH-COMPLEX – Analytical and Program Center for Advanced Environmentally-Friendly Technologies, Maria Curie-Skłodowska University, Głęboka 39, Lublin, 20-033 Poland; 6 Department of Polymer Chemistry, Institute of Chemical Science, Faculty of Chemistry, Maria Curie-Skłodowska University, M. Curie-Skłodowska Sq. 5, Lublin, 20-031 Poland; 7 Division of Wood Chemistry and Pulp Technology, Department of Fiber and Polymer Technology, School of Chemistry, Biotechnology and Health, KTH Royal Institute of Technology, Teknikringen 56-58, 100 44, Stockholm, Sweden

**Keywords:** Chemistry, Materials science, Nanoscience and technology

## Abstract

The growing demand for sustainable packaging, stricter regulations on non-biodegradable plastic waste, and increasing consumer awareness of environmental pollution are driving the development of water-soluble packaging materials. This study investigates the potential of lignin nanoparticles (LNPs) derived from spruce kraft lignin (SKL) and eucalyptus kraft lignin (EKL), as functional additives in polyvinyl alcohol (PVA)-based films to achieve an optimal balance between high transparency and effective UV protection. To improve LNP dispersion within the PVA matrix, hydrophobic domains were introduced into lignin via acetylation, as confirmed by ³¹P NMR spectroscopy. The morphology of the nanoparticles was analyzed using transmission electron microscopy (TEM). The resulting PVA–LNP nanocomposite films exhibited excellent transparency and outstanding UV-shielding capabilities. UV–Vis spectroscopy confirmed the UV-blocking performance of the films, revealing that EKL-derived nanoparticles (EKL-C1) enhanced UV absorption more than eightfold compared to neat PVA, while SKL-derived nanoparticles (SKL-C1) achieved a 6.5-fold increase. This superior performance can be attributed to the higher syringyl (S) unit content and abundant methoxy groups in EKL-C1, which can improve UV absorption efficiency. Atomic force microscopy (AFM) further demonstrated smoother surface morphologies for EKL-C1-containing films, indicating improved nanoparticle dispersion and reduced aggregation. Mechanical testing before and after UV exposure confirmed the suitability of the films for packaging applications. These findings highlight the potential of lignin-based nanocomposite films as eco-friendly packaging and coating materials, offering a unique combination of high transparency and robust UV protection while, providing valuable insights into the structure–property relationships of lignin nanoparticles in biodegradable polymer films.

**Supplementary Information:**

The online version contains supplementary material available at 10.1038/s41598-025-19753-6.

## Introduction

In recent years, water-soluble packaging has gained a particular attention among manufacturers of consumer goods. This trend is driven by increasing global consumer awareness of environmental issues such as plastic pollution and the accumulation of synthetic, non-biodegradable materials, as well as stricter government regulations on waste management^1^. According to a report by Future Market Insights, the water-soluble packaging market is predicted to reach US$ 4,786 million by 2033^2^, making it a key contributor to the circular economy with a strong emphasis on sustainability and waste reduction. Major companies are investing in research to diversify the application of water-soluble packaging materials and improve of their performance.

Currently, among water-soluble polymers, poly(vinyl alcohol) (PVA) is the most widely produced^3^. PVA is a hydrophilic, synthetic, biodegradable polymer obtained by polymerizing vinyl acetate into polyvinyl acetate, followed by hydrolysis of the acetate groups. It is one of the few vinyl polymers that are soluble in water and ultimately biodegradable^4^. In natural conditions, the hydroxyl groups in PVA are first oxidized to diketones, which are then hydrolized at the carbon‑carbon diketone bonds. In the final step, microorganisms degrade the polymer completely into to CO_2_ and H_2_O^5^. PVA is well known for its excellent film-forming and adhesive properties. Its main industrial applications include the textile industry, pharmaceuticals, and personal care products. It has also gained significant attention in the field of advanced multifunctional packaging, mainly due to its non-toxicity, biodegradability, and high transparency. The safety of PVA has been confirmed by the FAO/WHO Joint Committee of Experts on Food Additives^6,7^. After industrial use, PVA is typically transported via wastewater to treatment plants, where it undergoes degradation. However, when applied in agriculture, it tends to persist in the soil and exhibits low biodegradability^8^. Therefore, it is important to identify effective fillers that can enhance the properties of PVA, resulting in more environmentally friendly final material.

Lignin is a promising filler for PVA foils. As the second most abundant biomass resource in plants, it is relatively inexpensive and widely available^9^. Commercially, Lignin is a by-product of the pulp and paper industry, which produces more than 50 million tons of lignin annually. Most of it is burned as a low-value fuel to recover energy and chemicals – a necessary step in the kraft pulping process, which leads to severe resource underutilization. However, with the development of modern extraction technologies such as LignoBoost and LignoForce, it is now possible to isolate a portion of lignin from black liquor, making it increasingly available for value-added applications^10^.

Lignin possesses a unique chemical structure, rich in functional groups such as hydroxyl, methoxyl, carboxyl groups, carbonyl, and quinone groups. These functional groups enable its application in polymer materials, where it can impart valuable properties such as ultraviolet (UV) absorption, antibacterial activity and antioxidant properties^11^. Among these, UV protection is particularly deserves special attention^12–17^. Lignin’s excellent UV absorbing capacity is attributed to its aromatic rings, methoxy groups, and conjugated double bonds^18–20^.

Pure PVA has poor resistance to UV radiation and undergoes degradation upon exposure to UV light^21^, which compromises the structural integrity and long-term performance of PVA-based coatings. To enhance UV stability and extend material durability, lignin, particularly in the form of lignin nanoparticles (LNPs), can be incorporated into the PVA matrix due to its strong UV-absorbing capacity. In addition to improving UV resistance, the use of lignin contributes to the sustainable development of bio-based nanocomposites and supports biomass valorization by utilizing an abundant industrial byproduct^22–24^.

It is important to note that strong hydrogen bonding and π-π interactions within lignin can lead to agglomeration and irregular distribution of hydrophobic and polar groups in lignin aggregates. This, in turn, reduces the miscibility between lignin and PVA, potentially causing macroscopic phase separation. On the other hand, based on the observations provided by^25^, lignin is still capable of forming hydrogen bonds with PVA, even though the blend system remains immiscible. To improve miscibility between lignin and PVA, lignin must be homogeneously distributed within the PVA matrix. This can be effectively achieved by incorporating lignin in the form of nanoparticles^26,27^. This improves interfacial compatibility and enhances the barrier properties of the resulting composite. The improved interaction between the polymer matrix and lignin nanoparticles (LNPs) can be attributed to their increased specific surface area and abundance of surface-active sites, which facilitate better dispersion and integration within the host matrix^28,29^. Another key factor influencing miscibility is the structure of lignin, particularly the presence of hydroxyl groups. A higher content of ether linkages and hydroxyl groups can promote better miscibility with PVA^30–32^.

The novelty of this study lies in the use of acetylated lignin nanoparticles derivied from two distinct sources, spruce and eucalyptus, to develop transparent PVA foils with significantly enhanced UV-shielding properties. By improving nanoparticle dispersion through acetylation and establishing a correlation between lignin structure and UV absorption performance, this work offers new insights into the structure–function relationship in biopolymer composites. The comparative analysis, combined with a proposed UV-blocking mechanism, distinguishes this work from prior studies and highlights lignin’s potential as a sustainable additive for high-performance biodegradable packaging material.

## Materials and methods

### Materials

LignoBoost spruce kraft lignin (SKL) and LignoBoost eucalyptus kraft lignin (EKL), extracted from the corresponding black liquors using LignoBoost technology, were kindly provided in powder form (95% dry) by Stora Enso and Suzano pulp and paper companies. PVA was purchased from Sigma Aldrich (Mw 13,000–23,000, 87–89% hydrolyzed).

### Acetylation of lignin samples

The acetylation of lignin was carried out according to the method by^26^. Initially, 1 g of dried kraft Lignin, obtained from either Norway spruce or eucalyptus, was dispersed in 30 mL of pyridine until fully dissolved. Subsequently, 20 mL of acetic anhydride was gradually added in three equal portions at three-hour intervals after adding pyridine. The reaction mixture was continuously stirred at high speed for 30 h. Upon completion, the dark solution was Transferred into 500 mL of 0.1 N hydrochloric acid and left to precipitate in an ice bath for 30 min. The precipitate was then collected using a 0.45 μm nylon filter, thoroughly rinsed with distilled water, and left to dry on the filter at room temperature for 48 h.

### Synthesis of lignin nanoparticles (LNPs)

Acetylated lignin samples were dissolved in a mixture of acetone and water (4:1, v/v) to prepare a solution with a Lignin concentration of 1 mg/mL. To eliminate undissolved particles and potential aggregates, the solution was filtered through a 0.45 μm membrane filter. While stirring at a moderate speed, four volumes of deionized water were gradually added dropwise to the Lignin solution to induce nanoprecipitation. The mixture was then stirred continuously for two days, allowing the acetone to fully evaporate. The final Lignin concentration in the nanoparticle suspension was 0.24 mg/mL^33^.

### Preparation of foils

A total of 5.0 g of poly(vinyl alcohol) and 90 ml of distilled water were placed in a 250 ml three-necked flask equipped with a mechanical stirrer, a thermometer, and a reflux condenser. The mixture was stirred at room temperature for 15 min and then left to stand for 24 h. Subsequently, the contents of the flask were heated to 80–85 °C and stirred at 300 rpm for 3 h using a mechanical stirrer. The resulting clear aqueous PVA solution was diluted with distilled water to a final volume of 100 ml. The films were cast in Petri dishes with a diameter of 8 cm. The prepared samples were then placed in a dryer and left for 24 h. Increasing amounts of the solution of LNPs were incorporated into the PVA matrix, as shown in Table 1. The final thickness of the lignin/PVA films was approximately 0.3 mm on average.

**Table 1:** Composition of the pla/lnps nanocomposite foils.

Sample	PVA_aq_	SKL_aq_	EKL_aq_
	(g)		
PVA (Ref)	15	0	0
2SKL-C1	15	2	0
5SKL-C1	15	5	0
10SKL-C1	15	10	0
15SKL-C1	15	15	0
2EKL-C1	15	0	2
5EKL-C1	15	0	5
10EKL-C1	15	0	10
15EKL-C1	15	0	15

## Methods

The functional groups in lignin samples were quantified using ^31^P NMR spectroscopy, following established methods^34,35^. Around 28–32 mg of the original (SKL and EKL) or acetylated (SKL-C1 and EKL-C1) Lignin sample was dissolved in 100 µL each of DMF and pyridine. Endo-N-hydroxy-5-norbornene-2,3-dicarboximide (40 mg/mL) was used as an internal standard, and chromium (III) acetylacetonate (5 mg/mL) acted as a relaxation reagent. Phosphorylation was performed with 2-chloro-4,4,5,5-tetramethyl-1,3,2-dioxaphospholane, and the derivatized sample was dissolved in CDCl₃ before analysis. ^31^P NMR spectra were recorded using inverse-gated proton decoupling with a 90° pulse angle, a 10 s delay time, and 256 scans, with a total acquisition time of 29 min.

A transmission electron microscope (TEM) was utilized to examine the core-shell structure of lignin nanoparticles. Imaging was conducted using a Hitachi HT7700 series instrument (Hitachi, Japan) with an accelerating voltage of 100.0 kV and an emission current of 8.0 µA.

SEM micrographs were taken using an FEI Phenom-World scanning electron-ion microscope. The AFM images were recorded using the Veeco Nanoscope V microscope (USA) for the surface mapping of the fabricated materials and to calculate the average (R_a_) and the root mean square (R_q_) roughnesses.

Fourier transform infrared (FTIR) spectra of the films were obtained using a Thermo Nicolet 8700 FTIR spectrometer (Thermo Scientific, Waltham, MA, USA) equipped with a Smart Orbit™ diamond ATR and a DTGS (deuterated triglycine sulfate) detector. The DTGS detector ensured stable signal performance within the mid-infrared spectral range (4000–400 cm^−1^). After applying baseline correction, ATR adjustment, and scaled normalization, the processed spectra were equivalent to transmission spectra.

UV-Vis electronic absorption spectra of nanocomposite foils were recorded using a dual-beam UV-Vis Cary 300 Bio spectrophotometer (Varian), which includes a thermostatted tray for a 6 × 6 Peltier block. The instrument allows for measurements in different solutions as well as in polymer coatings, as was necessary for this study, using a specialized attachment designed for solid samples. Temperature regulation during the measurements was controlled by a thermoelectric probe (Cary Series II from Varian).

The UV-blocking efficiency of PVA and PVA-LNP composite films was assessed using a Shimadzu UV-2600 UV-vis spectrophotometer (Japan) equipped with an integrating sphere. The spectra of films, normalized to a thickness of 0.3 mm, were recorded over a wavelength range of 200–600 nm. All measurements were baseline-corrected using the empty sample compartment (air).

The average transmittance ($\:\stackrel{-}{T})$ was estimated from the Eq. (1):

1 $$\:\stackrel{-}{T}\, =\frac{{\int}_{{\lambda}_{1}}^{{\lambda}_{2}}T\left(\lambda\right)d\lambda}{{\lambda}_{2}-{\lambda}_{1}}$$

where T is measured transmittance.

The average transmittance of UVC (275–200 nm), UVB (320–275 nm), UVA (400–320 nm), and visible light (600–400 nm) was estimated^14^.

Color variation was assessed using a Konica Minolta C544e scanner^36^. The CIELAB color variables—L*, a*, and b*, as defined by the International Commission on Illumination (CIE, 1965)^37^ —were analyzed. Here, L* represents lightness, while a* and b* denote the red/green and blue/yellow color dimensions, respectively.

Polymer coatings were applied to a white standard plate and scanned. The obtained images were processed using Online Photoshop to extract x, y, and z color coordinates, which were then converted into L*, a*, and b* values using an online calculator^38^.

The color difference (ΔE*) among coatings was calculated using Eq. (2), PVA resin without lignin served as the control:

2 $$\:\varDelta{E}^{*}=\sqrt{{\left(\varDelta{L}^{*}\right)}^{2}+{\left(\varDelta{a}^{*}\right)}^{2}+{\left(\varDelta{b}^{*}\right)}^{2}}$$

Three random measurements were taken on the surface of each coating to ensure reproducibility.

Thermogravimetric analysis (TGA) was performed using a TGA/SDTA 851 METTLER TOLEDO apparatus. The thermal degradation characteristics of PVA and LNPs-containing PVA films were assessed by analyzing 5–10 mg of each sample. The measurements were conducted in a nitrogen atmosphere (50 ml/min) with a heating rate of 10 °C/min, covering temperature range from 25 °C to 800 °C.

The mechanical properties of the prepared films, tensile strength, Young’s modulus, and elongation at break, were measured using a Zwick Roell Z010 testing machine (Ulm, Germany).

To evaluate the impact of lignin incorporation on the environmental stability of the films, samples (50 mm × 10 mm × 0.3 mm; five replicates per composition) were subjected to accelerated aging in an Atlas Xenotest^®^ Alpha **+** chamber equipped with a xenon arc lamp and a daylight filter system to simulate natural sunlight. The exposure conditions were set to an irradiance of 60 W/m² (300–400 nm), chamber temperature of 38 °C, black standard temperature of 65 °C, relative humidity of 50%, and a total exposure time of 100 h, following ASTM G155. After aging, all samples were conditioned at 23 °C and 50% relative humidity for 24 h prior to mechanical testing to ensure consistent measurement of their mechanical performance.

## Results and discussions

### Properties of LNPs

To enhance the compatibility of lignin with the hydrophilic PVA matrix and improve its dispersion, acetylation was performed to reduce the hydroxyl content and increase the hydrophobicity of lignin. This modification is expected to alter the interfacial interactions within the composite system, potentially enhancing the material’s optical and mechanical properties^26^. The functional groups of lignins before and after acetylation were quantified using ^31^P NMR, and the results are presented in Table 2. The content of aliphatic hydroxyls (Aliph-OH), total phenolic hydroxyls (Ph-OH_tot_), condensed and non-condensed guaiacyl units (G-OH), and syringyl units (S-OH), as well as *p*-hydroxyl-OH in the lignin samples, were measured and calculated. Both lignins contain significantly higher amounts of Ph-OH_tot_ in comparison to Aliph-OH. SKL, derived from softwood, is characterized by higher G-OH content, which results in a highly branched and cross-linked structure. EKL, derived from hardwood, contains a significant amount of S-OH, which results in a more linear and less cross-linked structure^39^. SKL also contains a higher content of *p*-hydroxyl-OH. As a result of the modification, over 90% of aliphatic and phenolic hydroxyl groups were consumed during acetylation.

**Table 2:** Hydroxyl contents of original and acetylated lignin determined by ^31^P NMR.

	Aliph-OH	Ph-OH_tot_	Condensed phenolics	Non-condensed phenolics		
			Condensed G-OH	S-OH	Non-condensed G-OH	*p*-hydroxyl -OH
**SKL**	1.83	3.64	1.47	/	2.01	0.15
**SKL-C1**	0.10	0.18	0.09	/	0.07	0.02
**EKL**	1.47	4.24	1.16	1.96	1.02	0.09
**EKL-C1**	0.14	0.23	0.08	0.08	0.06	0.01

Nanoparticles from unmodified lignin tend to form larger, more aggregated particles due to their hydrophilic nature and strong intermolecular interactions (e.g., hydrogen bonding). In contrast, acetylated lignin results in smaller, more uniform particles with improved dispersion stability, particularly in organic solvents and non-polar polymer matrices. Nanoparticles from acetylated lignin have been prepared and investigated for a range of applications^40^ including in antifogging coatings and structural color films^41^, as a possible vehicle for photosensitizing molecules^42^, and as a biomarker^43^.

**Fig. 1:**
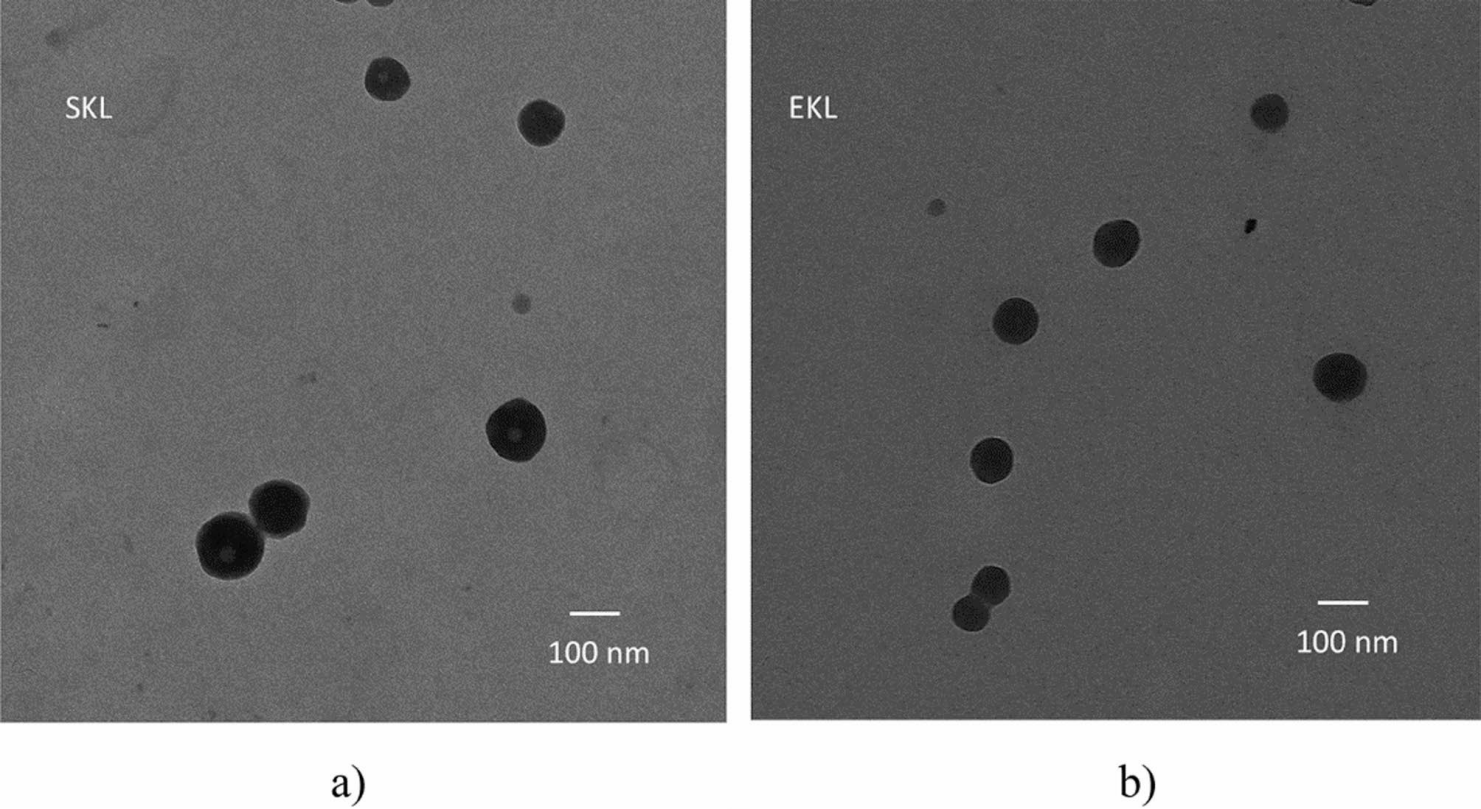
TEM micrograph of (**a**) SKL-C1 and (**b**) EKL-C1 nanoparticles.

The core–shell structure and well-defined spherical shape of the acetylated LNPs in this study were confirmed using TEM (Fig. 1). Minimal nanoparticle aggregation was observed, likely due to the consumption of hydroxyl groups during acetylation, which increases surface hydrophobicity and reduces hydrogen bonding. The uniform spherical structure and narrow size distribution of LNPs highlight their potential as sustainable nanofillers for the development of nanocomposite films.

## Properties of nanocomposite foils

### ATR/FTIR spectroscopy

**Fig. 2:**
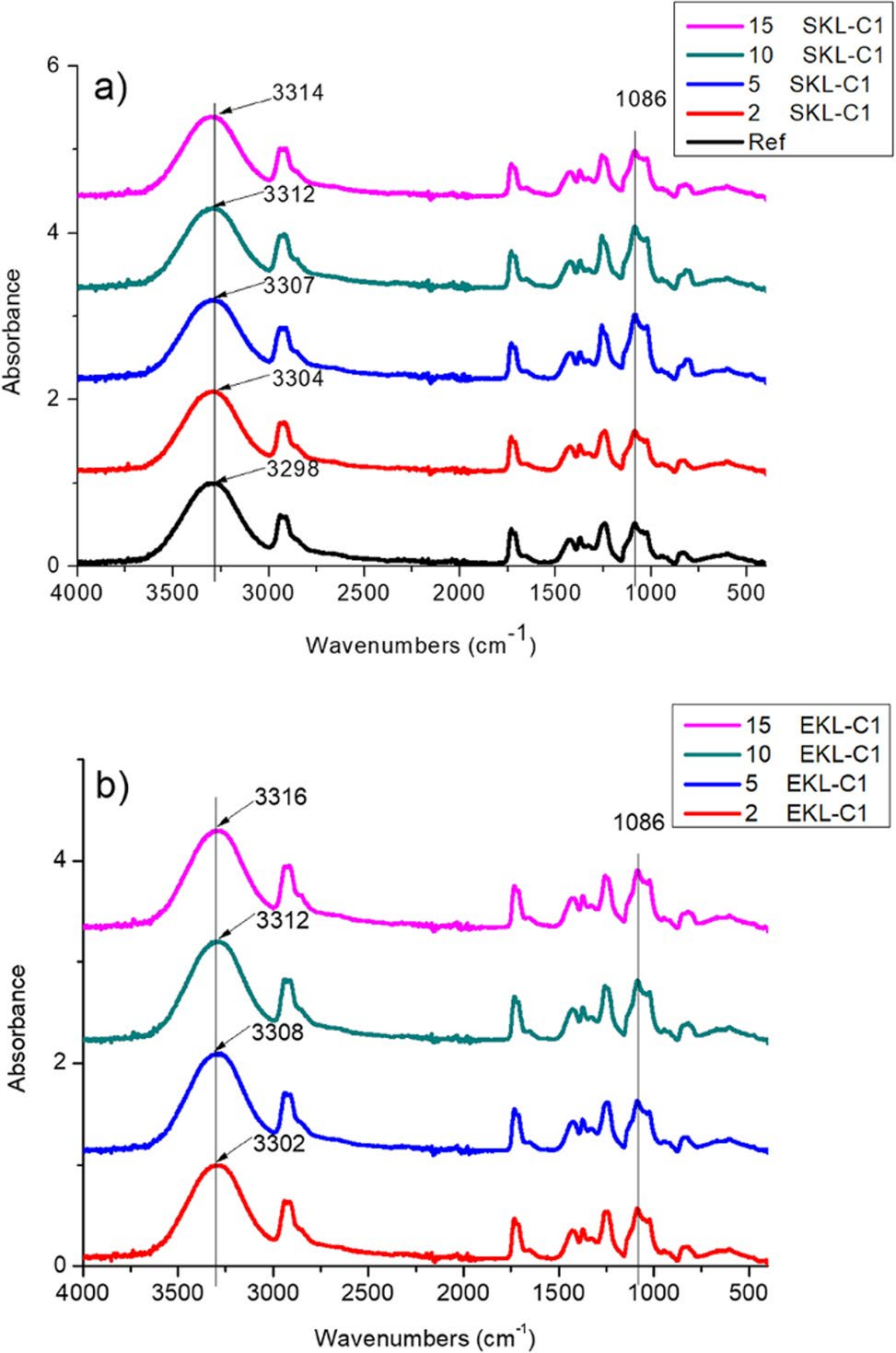
ATR-FTIR spectra of the nanocomposite foils with (**a**) SKL-C1 and (**b**) EKL-C1.

To investigate the interactions between the components of the foils, Fourier Transform Infrared Spectroscopy (FTIR) was used. Figure 2 presents the ATR-FTIR spectra of pure PVA film (Ref) and composite foils containing different amounts of LNPs from (a) spruce and (b) eucalyptus. The PVA films show a characteristic absorption band corresponding to the stretching of hydroxyl groups at 3298 cm^−1^^44^. A notable blue shift was observed with increasing LNP content in the nanocomposite foils, with the hydroxyl stretching band shifting from 3298 cm⁻¹ to 3314 cm⁻¹ for spruce-derived lignin (SKL) and to 3316 cm⁻¹ for eucalyptus-derived lignin (EKL) at the highest LNP concentrations. Additionally, the peak at 1086 cm^−1^ corresponding to C-O stretching vibrations changed slightly with the introduction of LNPs. The blue-shift phenomenon in FTIR spectroscopy refers to a shift towards higher vibrational energy frequencies, which is usually observed in hydrogen-bonded complexes^45,46^.

### SEM studies

**Fig. 3:**
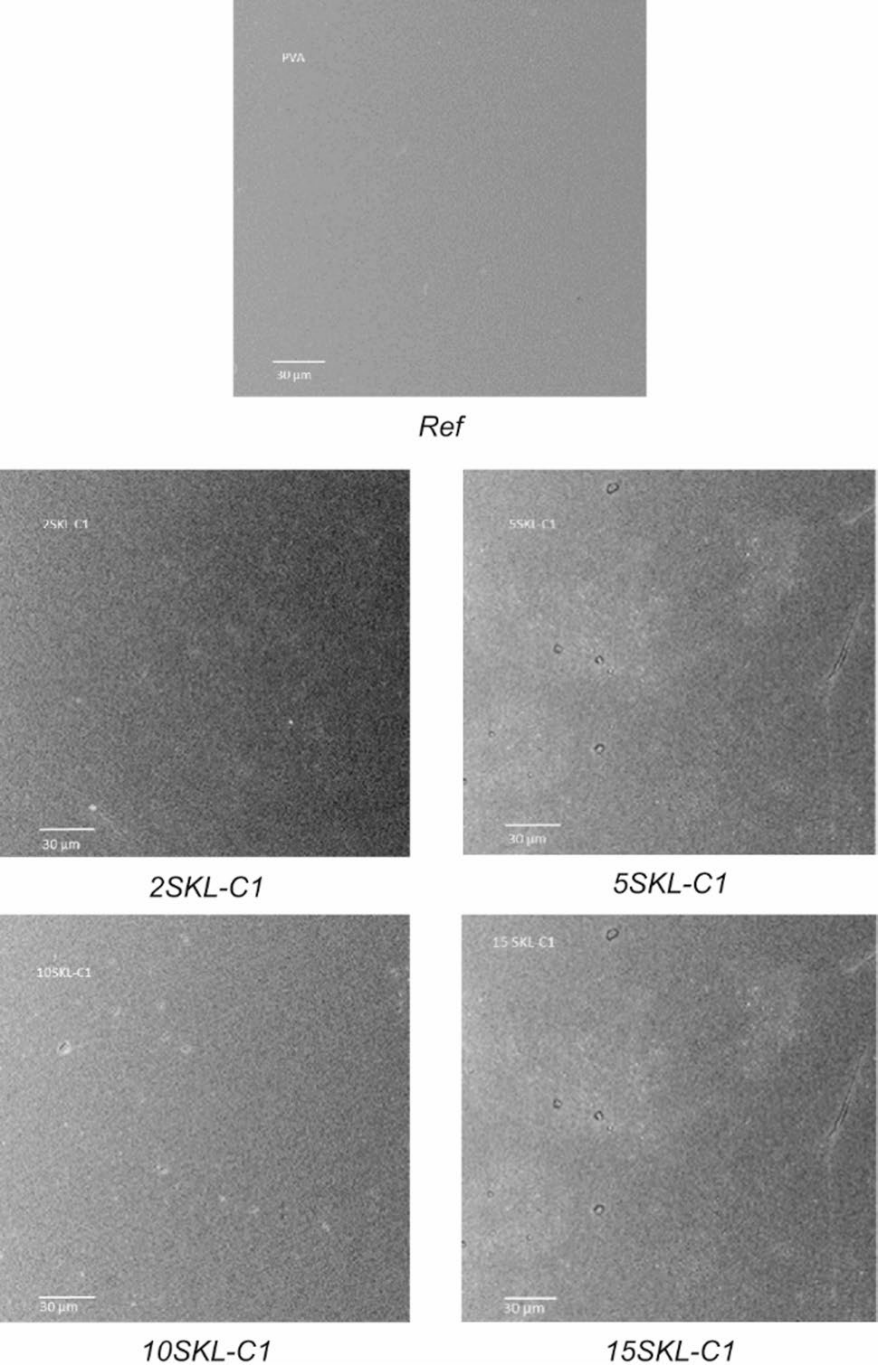
Figure SEM micrographs of the foils with nanoparticles prepared from spruce kraft lignin (SKL).

**Fig. 4:**
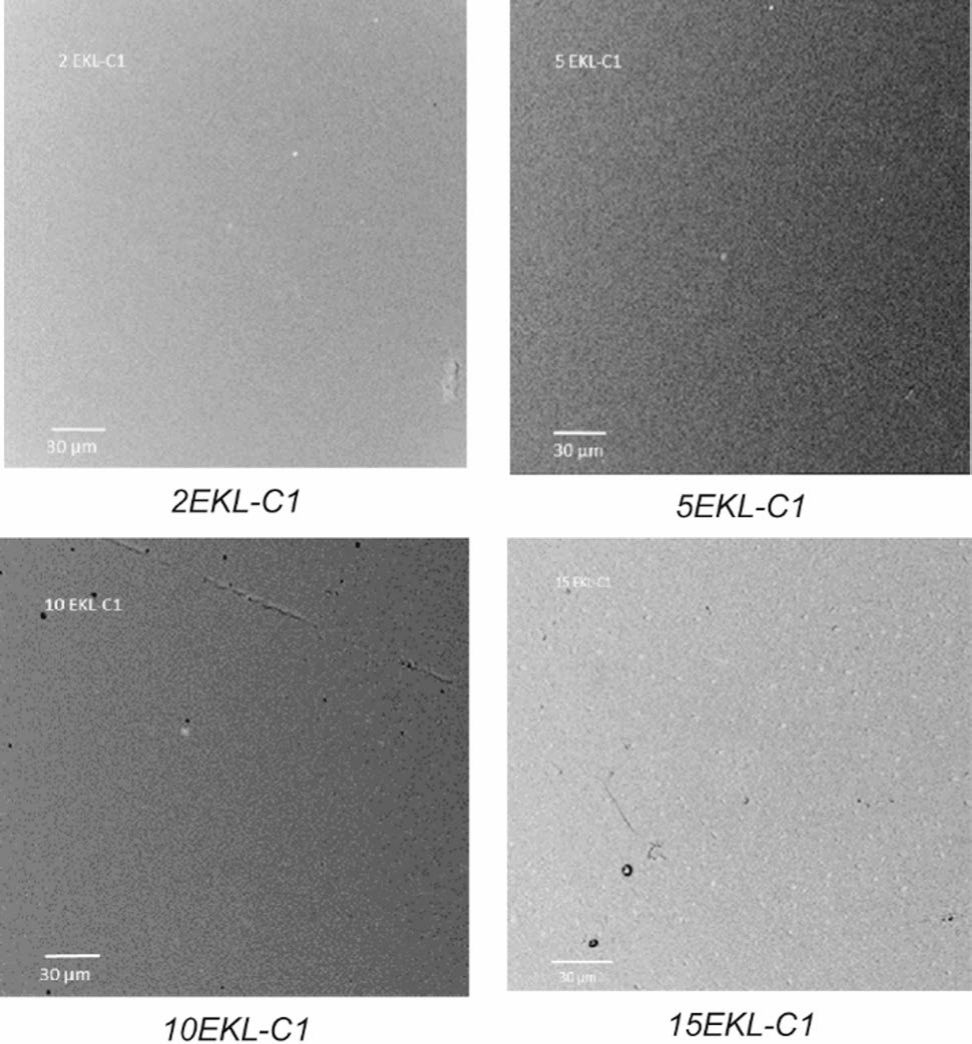
Figure SEM micrographs of the foils with nanoparticles prepared from eucalyptus kraft lignin (EKL).

SEM was used to examine the surface morphology of the tested samples at the microscopic level (Figs. 3 and 4). The reference sample exhibited the most continuous and uniform surface structure. In contrast, samples containing LNPs showed some irregularities, and more pronounced surface variations were observed in those incorporating SKL-C1 nanoparticles. These micro-scale changes may be a consequence of structural modifications occurring at the nanoscale. LNPs likely tend to agglomerate within the PVA matrix. Considering the origin of lignin, the steric arrangement of lignin molecules may influence the overall topography of the film.

### AFM analysis

**Fig. 5:**
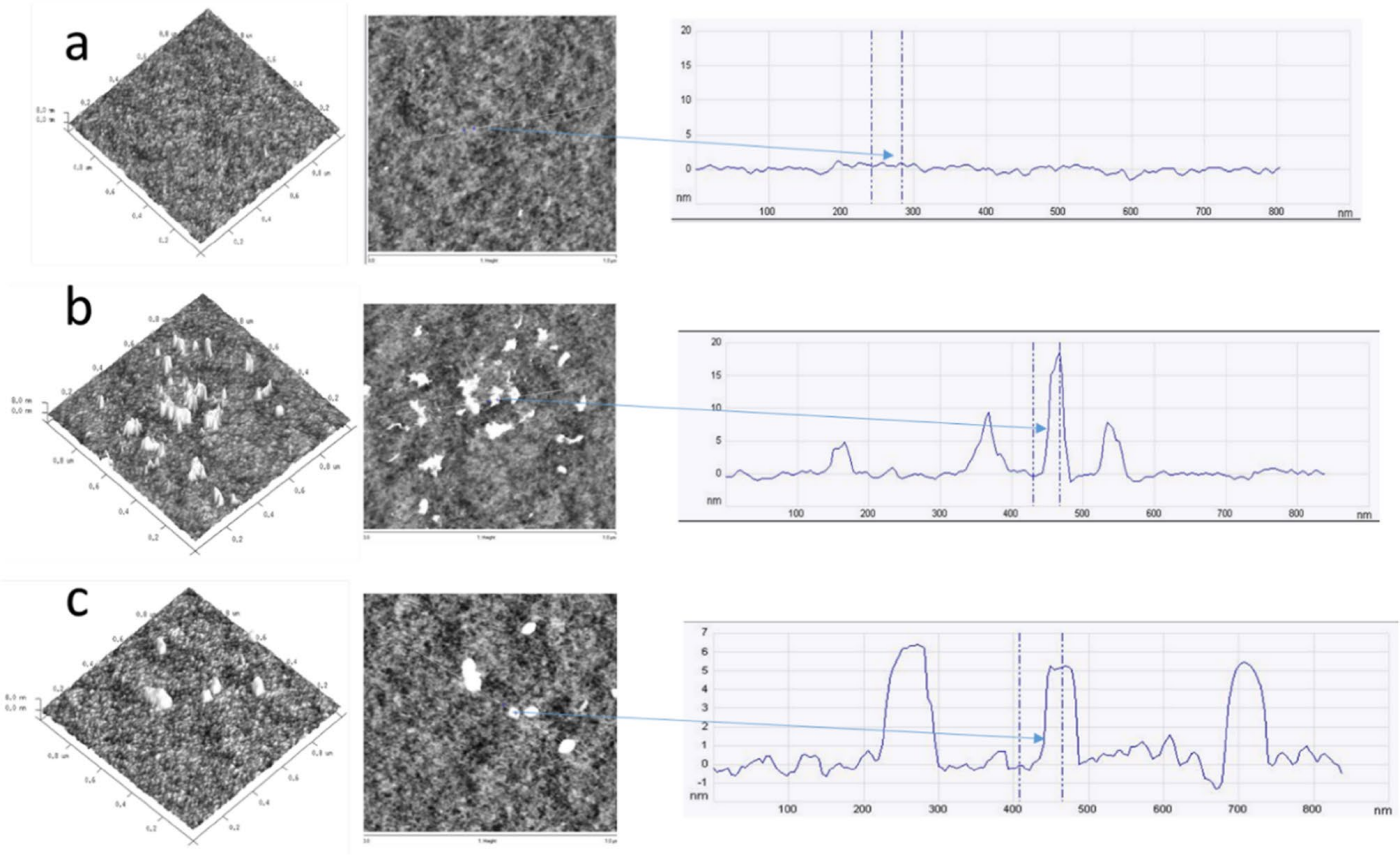
AFM images of (**a**) PVA foil, (**b**) PVA foil with 15SKL-C1, (**c**) PVA foil with 15EKL-C1.

To better understand the effect of LNPs addition on the surface topography of the obtained films, the AFM method was used, enabling observations at the nanoscale. The obtained images provide three-dimensional information, including the surface roughness parameters of the foils. AFM images are depicted in Fig. 5. The method used allowed us to confirm the presence of lignin nanoparticles on the surface of the tested materials and to determine the change in their roughness. The arithmetic roughness (R_a_) and the root-mean-squared roughness (R_q_) are common parameters used to describe roughness. The reference sample is characterized by smooth surface topography with R_a _= 0.411 nm and R_q_= 0.522 nm. For lignin-containing representative samples, the following roughness parameters were obtained: R_a_= 0.452 nm and R_q_= 0.791 nm (15EKL-C1) and R_a_= 0.647 nm and R_q_= 1.26 nm (15SKL-C1). Therefore, concerning the reference sample, a greater increase in the R_q_ than in R_a_ parameter was observed, which might indicate the presence of spikes on the surface of the tested samples. A different arrangement of lignin nanoparticles was also observed in the case of EKL-C1 and SKL-C1. Concerning EKL-C1, they had a rounded structure, while in the case of SKL-C1, they were more slender structures. This phenomenon may be related to the structure of EKL-C1, derived from hardwood, which contains a significant number of S-units, resulting in its more linear and less cross-linked structure and, therefore, lower roughness of the obtained foil. The roughness of the foil and the arrangement of the LNPs in the PVA matrix may be crucial for its functional properties, particularly concerning UV-shielding properties.

The discrepancy between TEM (spherical, ~ 100 nm particles) and AFM (irregular shapes, increased roughness) can be attributed to nanoparticle agglomeration and deformation during the film-forming process. SKL-C1 exhibits a greater tendency to aggregate due to its branched, guaiacyl (G)-unit-rich structure, whereas EKL-C1, with a more linear, syringyl (S)-unit-rich composition, shows improved dispersion and smoother surfaces, contributing to superior UV-shielding performance. The size and morphology of agglomerates further influence UV-blocking behaviour by affecting chromophore accessibility and light scattering, with smaller, well-dispersed particles (as in EKL-C1) enhancing both UV absorption and visible light transparency.

### UV-blocking performance of PVA-LNP foils

**Fig. 6:**
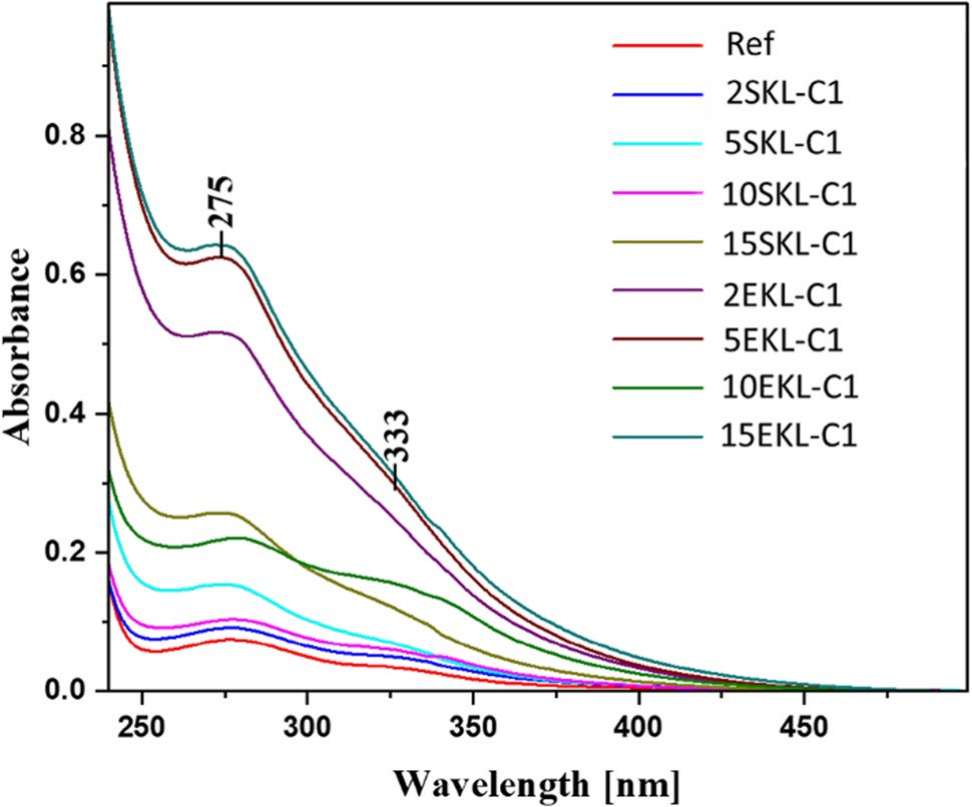
Electronic absorption spectra for the PVA film with the addition of spruce (SKL-C1) and eucalyptus (EKL-C1) lignin nanoparticles.

PVA–LNP composite films exhibiting superior UV-shielding performance hold significant potential for use in packaging applications^17^. Figure 6 presents the electronic absorption spectra of pure PVA (used as a reference) and nanocomposite foils containing lignin nanoparticles from acetylated softwood (SKL-C1) and hardwood (EKL-C1). The spectra, recorded in the 240 nm range and extending beyond the visible region, reveal clear difference depending on foil composition, primarily related to the type and concentration of LNPs used.

The solid red line in Fig. 6 corresponds to the UV-Vis spectrum of the pure PVA film, which matches Literature reports. The absorption increases with decreasing wavelength starting around 400 nm, with a pronounced peak at approximately 277 nm and an additional enhancement near 323 nm. According to the established Literature, the peak at 277 nm is attributed to the n→π* electronic transition of carbonyl groups in the polymer’s structure, while the enhancement near 330 nm corresponds to the π→π* electronic transition^47^.

Upon incorporation of SKL-C1 and EKL-C1 nanoparticles into polymer matrix, a clear increase in absorbance was observed. The extend of light absorption depended primarily on the type of nanoparticles used, as well as their concentration. Notably, the addition of the EKL-C1 nanoparticles resulted in higher absorbance increase compared to the corresponding additions of SKL-C1. In both cases, the adsorption bands with maxima around 275 and ~ 330 nm were significantly enhanced. This effect is attributed to the presence of lignin, whose molecular structure includes functional groups capable of both the n→π* and π→π* electronic transitions (Fig. 6). The highest levels of light absorption were, as expected, observed in samples containing the highest concentration of nanoparticles. It should be noted, however, that the most effective absorption was recorded in foil containing EKL-C1 nanoparticles. In fact, the third concentration level of the EKL-C1 already produced higher absorbance than the fourth concentration level of SKL-C1 (Fig. 6). This phenomenon, consistent with previous studies^36^, is attributed to the unique structural characteristics of lignin. The incorporation of lignin nanoparticles from both spruce and eucalyptus enabled the foils efficiently absorb UV light, in line with our initial assumptions.

Additionally, the spectra for both types of nanocomposites foils showed only minimal extension into the visible light region, indicating a relatively sharp boundary for photoprotective activity. Lignin inherently contains a variety of functional groups, such as hydroxy (*phenolic and aliphatic*), methoxy, carbonyl, and carboxy groups, which form extensive chromophoric systems capable of absorbing both UV and visible and light. In the UV range specifically, its absorption ability is primarily attributed carbonyl groups (C=O) and aromatic rings^48^. The extensive conjugation in lignin molecules enhances this effect, establishing lignin as an effective natural UV-blocking agent when incorporated into polymer matrices^49,50^. In the present study, the addition of lignin nanoparticles significantly enhanced the UV absorption capacity of the base PVA polymer, delivering improved photoprotection^51^.

In summary, UV-Vis spectroscopy revealed that the addition of spruce and eucalyptus Lignin nanoparticles had a significant and measurable impact on the photoprotective properties of the analyzed materials, particularly in the UV range. Compared to pure PVA, samples containing eucalyptus Lignin nanoparticles showed more than an 8-fold increase in UV Light absorption, while those with spruce Lignin nanoparticles showed an approximately 6.5-fold increase within the tested concentration range. These results highlight the strong potential of lignin nanoparticles, especially EKL-C1, as effective additives for enhancing UV protection in polymer films, supporting their applicability for commercial use in UV-sensitive packaging solutions.

**Fig. 7:**
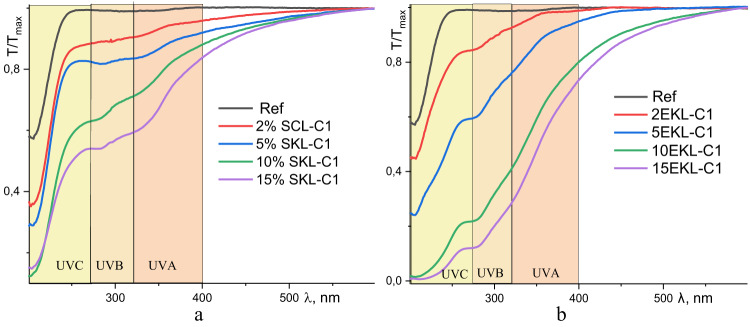
Normalized transparency spectra* of PVA films without (Ref) and with varying concentrations of LNP SKL-C1 (**a**) and EKL-C1 (**b**). *T/Tmax is used to express the transmittance at each wavelength relative to the maximum transmittance, which allows for a standardized comparison of light transmission across different samples and wavelengths.

Taking into consideration different regions of UV radiation, the UV-Vis transmittance spectra of PVA and PVA-LNP foils are additionally presented in Fig. 7.

As previously mentioned, pure PVA (Ref) foil exhibited high Transmittance across the UV spectrum, indicating a lack of inherent UV-blocking properties. However, the incorporation of LNPs significantly reduced Light Transmittance, particularly in the UV region. Both EKL- and SKL-based PVA foils demonstrated notable UV protection. At 2% LNP content, the composite foils partially shielded the UVA range (400–320 nm) and effectively blocked most of the UVB (320–275 nm) and UVC (275–200 nm) regions for both types of LNPs. As the LNPs content increased, UV-shielding efficiency improved, with foils containing 15% LNP-C1 effectively blocking most UVA radiation and fully shielding the UVB and UVC spectra.

**Fig. 8:**
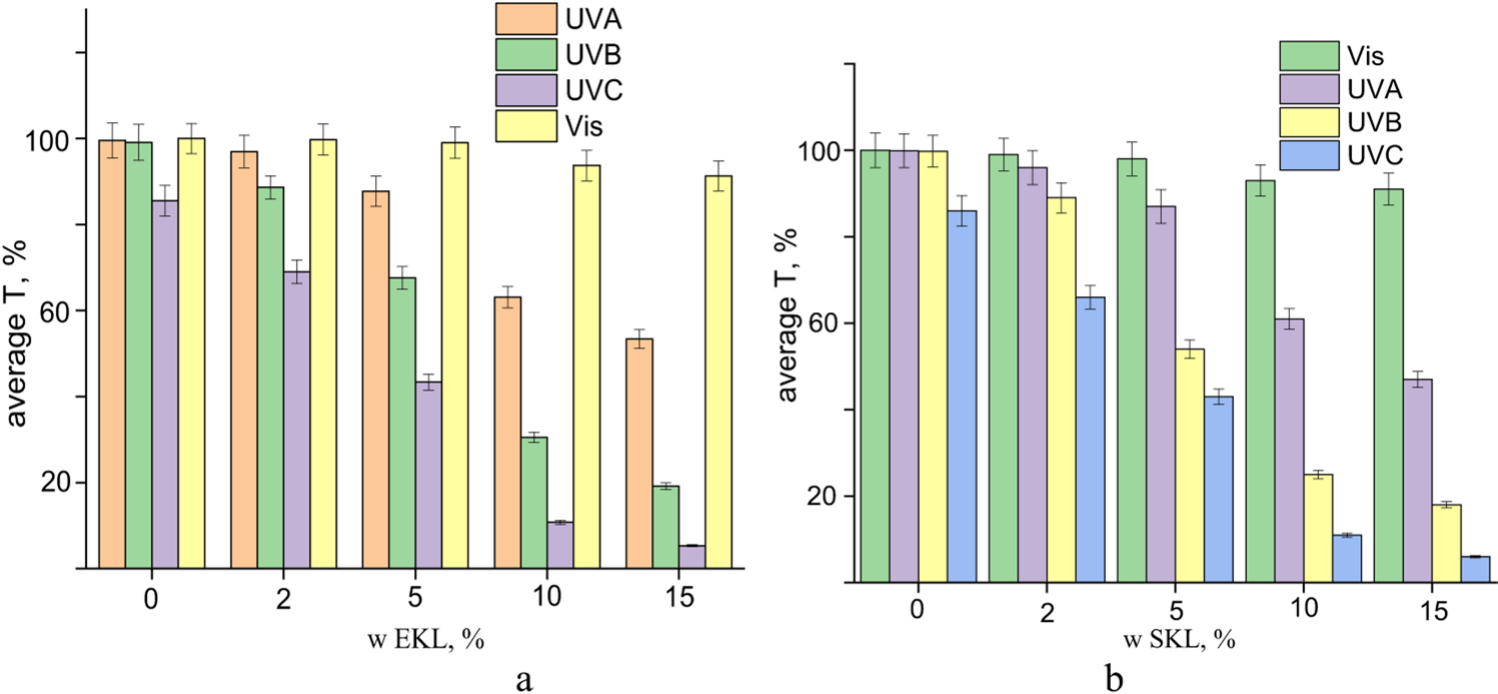
Protective properties of EKL-C1 (**a**) and SKL-C1 (**b**) across different regions of the UV spectrum.

**Fig. 9:**
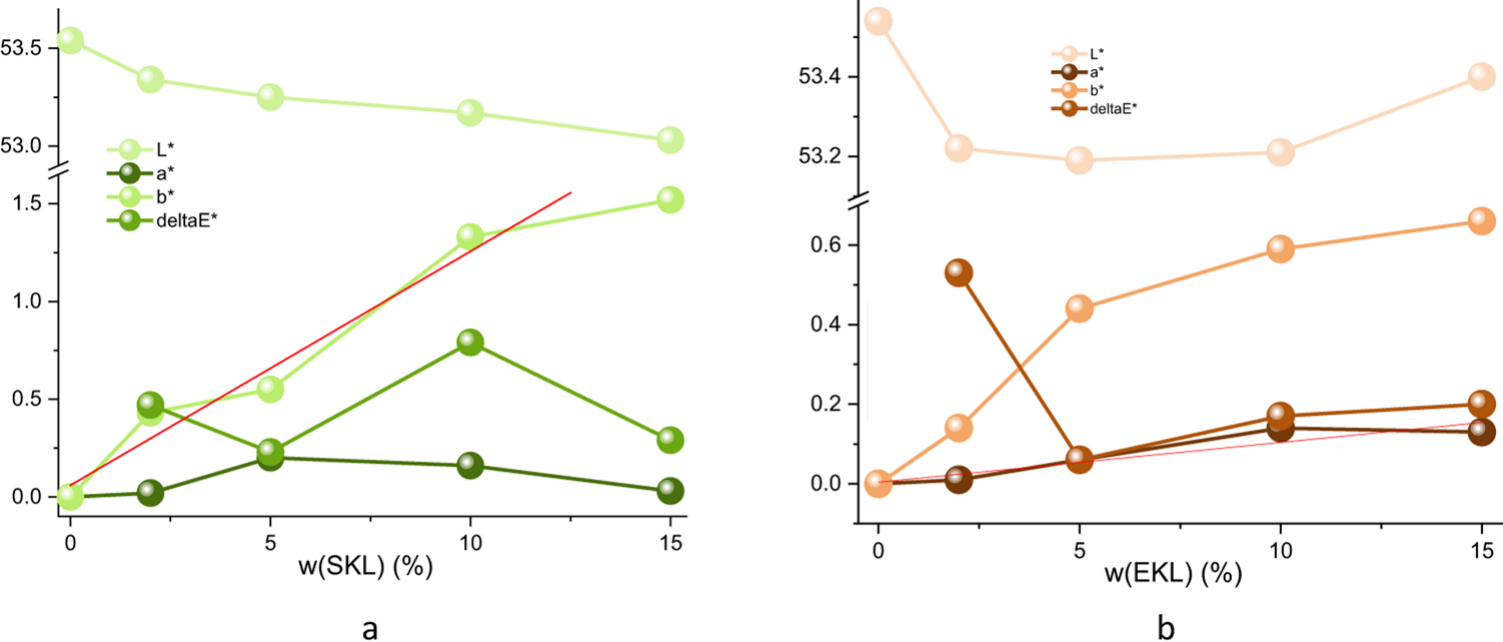
The influence of LNP content on color characteristics of LNP-PVA samples containing: **a**) SKL-C1, **b**) EKL-C1.

Figures 8 and 9 highlight the superior UV-shielding ability of foils containing nanoparticles from EKL-C1 compared to those containing SKL-C1. This enhancement can be attributed to the presence of syringyl units in EKL, which are rich in the methoxyl groups – functional moieties known to improve UV resistance (Table 2)^39,52^.

According to^53^, the shielding efficiency at characteristic wavelengths of 365 nm and 550 nm serves as a reliable metric for quantifying light-filtering capabilities (Figure S1). Similarly, studies^54,55^ have attributed the superior anti-UV performance of composite films to the oxygen-containing groups attached to aromatic rings in LNPs. Despite the light yellow coloration introduced by LNPs (Table S1), the composite foils maintained good transparency in the visible spectrum (λ > 400 nm), with Transmittance not falling below 74% for SKL-C1 and 69% for EKL-C1 (Figure S2). Variations in light absorption properties likely due to the interplay between nanoparticle concentration and dispersion, without significantly compromising optical clarity.

The degree of nanoparticle agglomeration within the PVA matrix significantly affects the UV-shielding properties of the foils. As shown by TEM and AFM analyses, EKL-C1 nanoparticles exhibit better dispersion and lower surface roughness compared to SKL-C1, which tends to form larger aggregates due to its branched, G-unit-rich structure. This improved distribution of EKL-C1 enhances chromophore accessibility and reduces light scattering losses, contributing to superior UV absorption. These findings highlight the importance of controlling nanoparticle aggregation during film formation to optimize the UV-protective performance of lignin-based PVA composites for sustainable packaging applications.

### Colorimetric properties

The addition of lignin nanoparticles affected the color properties of the nanocomposites. As shown in Table 3, increasing SKL content resulted in higher yellowness (b*) and reduced whiteness (L*). Conversely, EKL-C1 series foils exhibited a trend of increasing redness (a*) with higher EKL content. These differences align with the distinct properties of SKL and EKL, as noted in prior studies^56,57^. Specifically, EKL LNPs are primarily composed of S/G-units and carbohydrates, whereas SKL LNPs have a lower carbohydrate content and are rich in aliphatic side chains.

**Table 3:** Color characteristics of LNP-PVA.

LNP	L*	a*	b*	ΔE*
SKL-C1				
0	53.54	0	0	
2	53.34	0.02	0.43	0,47
5	53.25	0.20	0.55	0,23
10	53.16	0.16	1.33	0,79
15	53.03	0.03	1.52	0,29
EKL-C1				
0	53.54	0	0	
2	53.22	0.01	0.42	0,53
5	53.19	0.06	0.44	0,06
10	53.21	0.14	0.59	0,17
15	53.40	0.13	0.66	0,20

Minimal changes in film color were observed with LNP addition, regardless of lignin type (Table S1). Slight trends toward increased yellowness were detected for both SKL-C1 and EKL-C1 series as nanoparticle content increased, confirming the suitability of these composites as environmentally friendly protective coatings for food packaging applications.

Figure 9 reveals a linear relationship between LNP content and color parameters: *r* = 0.95 for a* (SKL-C1) and *r* = 0.99 for b* (EKL-C1). These correlations may enable rapid monitoring of LNP content during manufacturing.

### Thermal studies

**Fig. 10:**
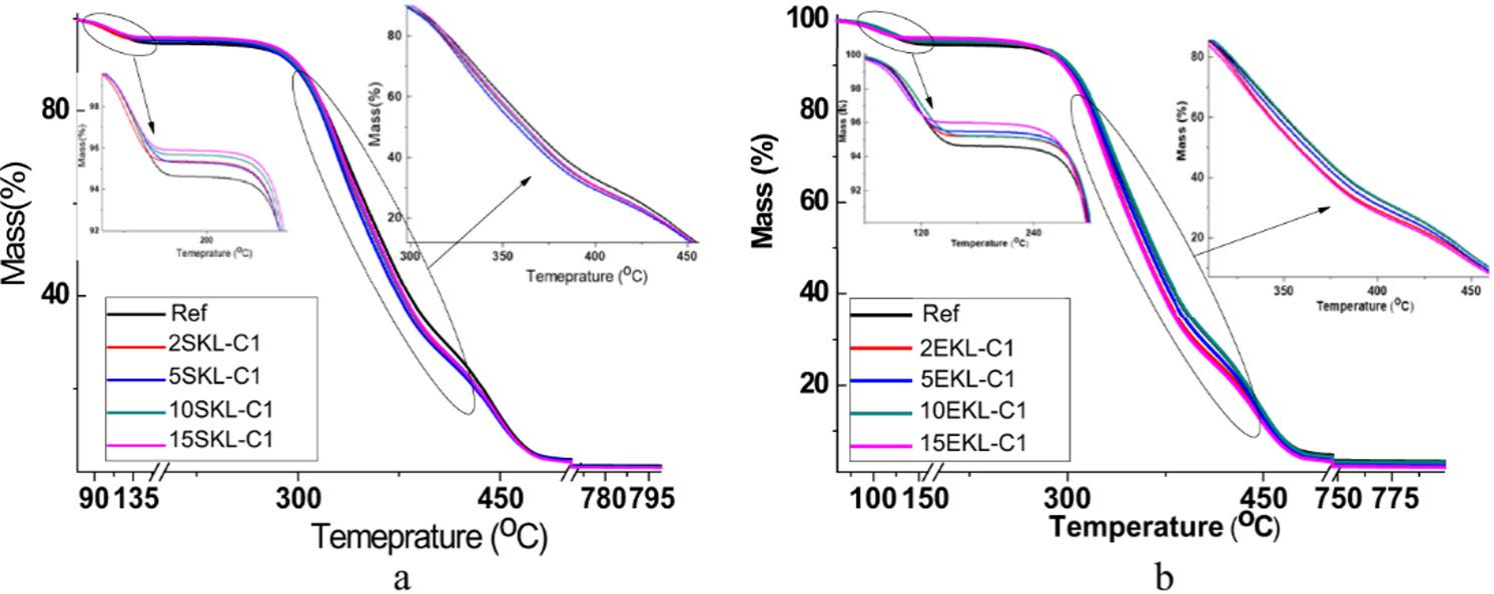
TG curves of the nanocomposite foils with SKL-C1 (**a**) and with EKL-C1 (**b**).

**Fig. 11:**
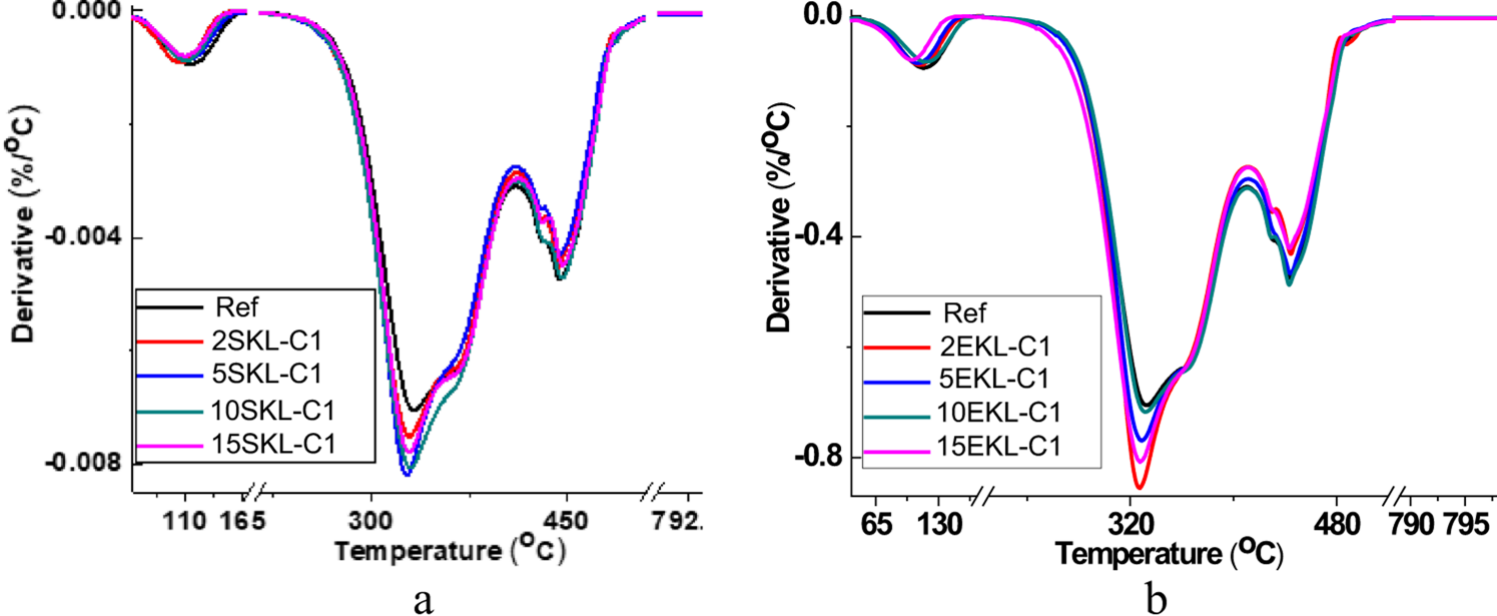
DTG curves of the nanocomposite foils with SKL-C1 (**a**) and with EKL-C1 (**b**).

Thermal resistance of the coating materials plays an important role in determining their usability. The thermal stability of the obtained foils was studied by means of thermogravimetric analysis. TG and DTG curves of the reference sample, nanocomposite foils with SKL-C1 and EKL-C1, are presented in Figs. 10 and 11. It is visible that pure PVA and LNP-containing PVA films show a similar trend of weight loss. For all the tested samples, three regions of mass loss are visible. The first stage, with the temperature of maximum thermal decomposition of 100 °C, is related to the loss of moisture or low molecular weight compounds. The second and major mass loss region is related to the structural degradation of PVA, mainly involving the dehydration of hydroxyl groups and the formation of volatile organic compounds and conjugated, unsaturated polyenes. In the third mass loss region (starting at around 395 °C in this study, with a maximum degradation rate at about 430 °C), polyene residues degrade into alkenes and alkanes, as well as aromatics through intramolecular cyclization^58–61^. The introduction of LNPs (especially EKL-C1) slightly influences the temperature with the fastest degradation rate (T_max_). Lignin addition can be beneficial due to its charring ability and therefore increasing fire resistance of polymer coating materials. Additionally, good thermal stability as well as hydrogen bonding between LNPs and PVA that can restrict the free movement of PVA chain segments^62^ characterize the presence of aromatic structural units in LNP molecules.

### Mechanism of UV blocking

The UV-blocking mechanism of PVA-LNP foils primarily relies on absorption rather than reflection or scattering, as supported by experimental data and previous studies^45^. LNPs absorb ultraviolet photons, converting their energy into heat through hydrophilic chromophores such as methoxy, phenolic hydroxyl, carbonyl, and carboxyl groups present on the nanoparticle surface^50^. The generated heat is gradually dissipated from the nanocomposite films without degrading the PVA matrix.

A slight increase in light transmittance at the UVC spectrum (Fig. 8) suggests that UV blocking is not influenced by particle size (e.g., via reflection), as acetylated SKL and EKL nanoparticles have similar size and shape (Fig. 1). Instead, the chromophoric groups within lignin molecules are likely responsible for UV absorption, as indicated by the correlation between increased yellowness (b*) and reduced UV transmittance (Figs. 8 and 9).

According to RGB color theory, yellow and blue light combine to produce white light when their intensities are balanced^63–65^. In this context, lignin nanoparticles function as yellow pigments, selectively absorbing blue light and regulating high-energy visible light transmission. As a result, the nanocomposite films remain opaque to UV light (λ < 400 nm) while maintaining high transparency in the visible spectrum (λ = 400–600 nm).

### Mechanical properties

**Fig. 12:**
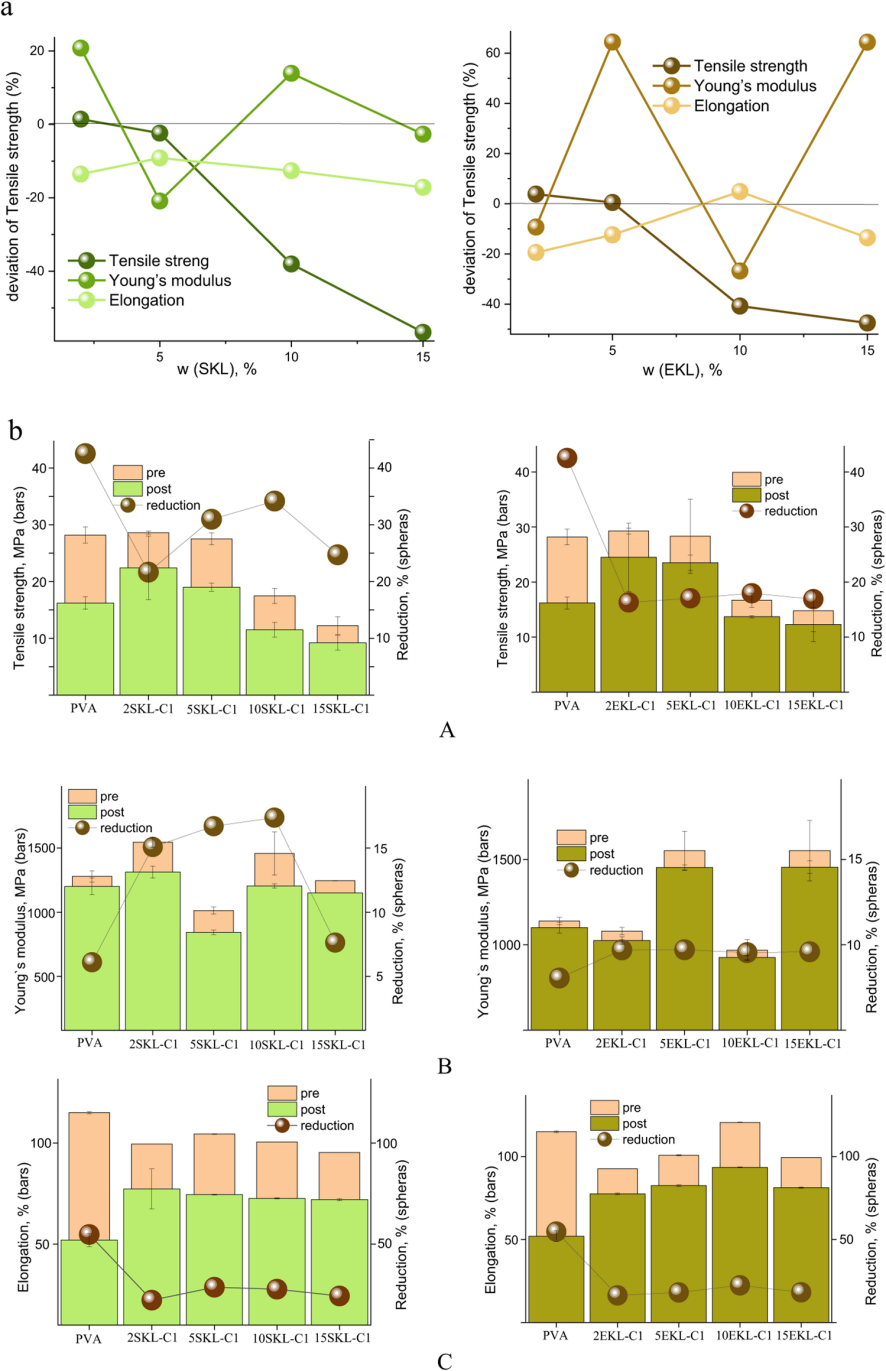
(**a**) Effect of Lignin nanoparticle content on the mechanical properties of PVA foils. (**b**) Effect of UV irradiation on the mechanical properties of lignin-containing foils.

Since the foils proposed in this study are intended for use as food packaging, it is essential to assess their mechanical strength to verify their ability to withstand the stresses and loads that arise from environmental factors and packaged products. The mechanical properties of foils enriched with lignin nanoparticles (LNPs) derived from spruce (SKL-C1) and eucalyptus (EKL-C1) at various concentrations were compared with those of the reference PVA foil (without nanoparticles). The results are summarized in Fig. 12a and b as well as in Table S2.

The incorporation of lignin nanoparticles, regardless of their source, had only a limited impact on the elongation at break of the foils. For SKL-C1 containing foils, increasing the Lignin content from 2 to 15 wt% resulted in a nearly Linear decrease in elongation, ranging from approximately 9% to 17%. In contrast, EKL-C1 containing foils exhibited a non-linear trend: elongation increased at intermediate concentrations and reached a maximum at 10 wt%, exceeding the value of the reference sample by 4%. These differences suggest distinct interactions between the two types of lignin nanoparticles and the PVA matrix.

The Young’s modulus of the foils fluctuated within the range of − 21% to + 21% for SKL-C1 and − 26% to + 64% for EKL-C1 relative to the reference film. These variations may reflect uneven dispersion of nanoparticles within the polymer matrix. Nevertheless, the overall trend toward increased modulus, particularly pronounced for EKL-C1, suggests that LNP incorporation generally enhances the stiffness of the polymer, implying a reduced flexibility with higher lignin content.

A gradual decline in tensile strength was observed with increasing nanoparticle content in both SKL- and EKL-based foils. This trend is consistent with previous findings^66^ and may be attributed to structural irregularities caused by LNP agglomeration. Despite this reduction, all measured values remained within the acceptable range for food packaging applications.

Ultraviolet (UV) irradiation is a well-known cause of photochemical damage and degradation in both food products and packaging materials. Packaging with UV-protective properties can help maintain food quality, enhance safety, and extend shelf life. Lignin nanoparticles (LNPs) are established UV-absorbing fillers, and their incorporation into PVA foils in this study aimed to enhance UV resistance. However, UV exposure can also lead to degradation of the polymer itself^67^. To assess the durability of the developed foils, mechanical properties were evaluated before and after UV exposure.

As shown in Fig. 12b, all tested parameters, tensile strength, Young’s modulus, and elongation at break, decreased following UV in all samples. This outcome is consistent with UV-induced polymer degradation. Nonetheless, the LNP-containing foils demonstrated greater resistance to UV-related deterioration compared to the reference PVA film, highlighting the protective role of lignin nanoparticles^68^.

**Fig. 13:**
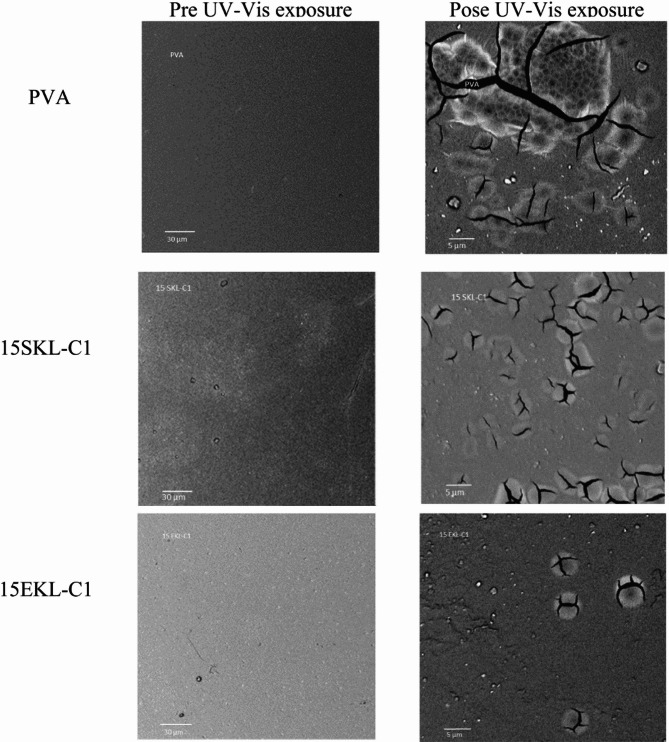
SEM micrographs of the foil samples before and after UV exposure.

Further insights were gained from SEM micrographs of representative foils (PVA, 15SKL-C1, and 15EKL-C1) before and after UV exposure (Fig. 13). The reference foil exhibited a higher number and larger size of cracks following irradiation. In contrast, the SKL-C1- and EKL-C1-containing foils showed significantly fewer cracks, indicating that both types of LNPs contribute to enhanced durability and reduced photodegradation. Notably, SKL-C1 foils displayed more pronounced cracking than EKL-C1 foils, suggesting that EKL nanoparticles offer superior reinforcement, likely due to their better dispersion (TEM, Fig. 1b) and smoother surface morphology (Ra = 0.452 nm, Fig. 5).

Taken together, these findings confirm the potential of PVA–LNP composite films, particularly those containing EKL-C1, for use in flexible food packaging applications. Their balanced mechanical properties, combined with improved UV stability, highlights their suitability for protecting food products while maintaining structural integrity during storage and handling.

### Barrier properties of PVA and LNP-enhanced PVA foils

An essential criterion in the selection of food packaging systems is their barrier performance, which determines the material’s ability to resist the transmission of gases (O₂, CO₂, and N₂), water vapor, light, and aroma compounds. These barrier properties are critical for maintaining the quality, safety, and shelf life of packaged products by preventing spoilage, contamination, and degradation. Poly(vinyl alcohol) (PVA) is well known for its excellent gas barrier properties, chemical resistance, and favorable optical and physical characteristics, highliting its strong potential as a biodegradable high-barrier coating for food packaging films^69^. However, PVA’s high sensitivity to moisture can significantly compromise its barrier performance, particularly under conditions of elevated humidity or in applications involving fresh foods^70^.

To address this limitation, kraft lignins have emerged as promising additives due to their intrinsic hydrophobic properties, which can reduce the water sensitivity of PVA films and improve their water vapor permeability (WVP), as supported by several studies^32,71^. Barrier parameters such as WVP and oxygen permeability (OP) are critical metrics, as they define the material’s ability to protect contents from external environmental factors. Previous studies have reported that the WVP of neat PVA films ranges from (9.9 × 10⁻⁴ to 4.9 × 10⁻⁴ g mm⁻¹ day⁻¹ atm⁻¹)^72^, while the OP of pure PVA has been measured at 0.12 ± 0.04 cm² m⁻² atm⁻¹ day⁻¹ MPa^73^.

The incorporation of lignin nanoparticles (LNPs), particularly acetylated types such as SKL-C1 and EKL-C1, is expected to improve water vapor barrier properties by reducing the hydrophilicity of the composite film. For example, Zhang et al. (2020)^32^ demonstrated that incorporating 5 wt% lignin into PVA reduced the water vapor transmission rate (WVTR) by approximately 189% compared with neat PVA. Similarly, Yang et al. (2024)^74^ reported that pure PVA films exhibited a WVTR of 6.9 g∙m⁻²∙h⁻¹ and a WVP of 3.24 × 10⁻⁷ g∙m m⁻² Pa h⁻¹ The addition of lignin nanoparticles led to a moderate reduction in WVTR. However, they also observed that excessive LNP content could lead to particle aggregation, disrupting uniform dispersion within the matrix and slightly increasing WVP values due to reduced densification and water retention capacity.

The source of lignin also plays a crucial role in determining barrier improvements. According to Lin et al.^39^, hardwood lignins (e.g., EKL) generally deliver superior barrier properties compared to softwood lignins (e.g., SKL) due to their more linear molecular structure and higher methoxyl group content, which further decreases water affinity. In addition to improving water vapor resistance, lignin nanoparticles enhance oxygen barrier properties by creating tortuous diffusion pathways and reinforcing the polymer matrix through hydrogen bonding. Phansamarng et al. (2024)^75^ confirmed that increasing lignin loading in films consistently reduced oxygen permeability, thereby strengthening their protective function against oxidative degradation in packaged foods.

Collectively, literature findings underscore that lignin nanoparticles, particularly those derived from hardwood-derived sources such as EKL-C1, significantly enhance the barrier performance of PVA-based films by reducing both WVP and OP. These results align with the present study, confirming the potential of acetylated SKL-C1 and EKL-C1 nanoparticles to improve the barrier properties of PVA foils and support the development of sustainable, high-performance food packaging materials.

## Summary of key findings

A detailed comparison of PVA foils containing lignin nanoparticles from spruce (SKL-C1) and eucalyptus (EKL-C1) is presented in Table 4. EKL-C1-based foils consistently outperformed SKL-C1 foils in UV-shielding efficiency, surface smoothness (AFM), and nanoparticle dispersion (TEM). Both types of lignin underwent extensive acetylation (> 90% hydroxyl group conversion), but EKL-C1 showed slightly lower residual hydroxyl content, indicating a marginally higher degree of acetylation.

These differences likely contributed to the improved compatibility of EKL-C1 nanoparticles with the hydrophilic PVA matrix, resulting in less agglomeration and more homogeneous dispersion in the films. Additionally, the syringyl-rich structure of EKL, with its higher methoxyl group content, is known to enhance UV absorption. These combined factors explain the superior UV-blocking and structural properties observed for EKL-C1-based foils.

Colorimetric and mechanical tests further confirmed that both SKL-C1 and EKL-C1 composites retained high transparency and flexibility, with EKL-C1 films showing slightly greater resistance to UV-induced degradation. Overall, these findings highlight the potential of acetylated hardwood-derived lignin nanoparticles as effective functional additives for biodegradable, light-protective packaging materials.

**Table 4:** Comparison of PVA foils containing SKL-C1 and EKL-C1 lignin nanoparticles.

Property	Pure PVA (Ref)	PVA with SKL-C1 (Spruce)	PVA with EKL-C1 (Eucalyptus)	Significance
UV Transmittance (UVA/UVB/UVC)	High transmittance (poor UV blocking)	Partial UVA shielding, near-complete UVB/UVC blocking at 15% LNP	Near-complete UVA/UVB/UVC blocking at 15% LNP	EKL-C1 foils provide superior UV shielding, especially in UVA, critical for packaging light-sensitive materials
Surface Roughness (AFM)	Ra = 0.411 nm, Rq = 0.522 nm	Ra = 0.647 nm, Rq = 1.26 nm at 15% LNP	Ra = 0.452 nm, Rq = 0.791 nm at 15% LNP	EKL-C1 foils have smoother surfaces due to better dispersion, reducing aggregation and enhancing material quality
Colorimetric Properties (CIELAB)	L* = 53.54, a* = 0, b* = 0	Increased yellowness (b* = 1.52 at 15% LNP), L* = 53.03	Increased redness (a* = 0.13), slight yellowness (b* = 0.66 at 15% LNP), L* = 53.40	Minimal color change with EKL-C1, better aesthetic suitability for packaging; linear correlation (*r* = 0.99 for b* in EKL-C1) enables manufacturing control
LNP Dispersion (TEM)	N/A	Uniform spherical nanoparticles, some aggregation	More uniform, minimal aggregation	EKL-C1’s linear structure (S-units) reduces aggregation, improving compatibility with PVA matrix
Thermal Stability (TGA)	Three-stage degradation (100 °C, ~ 395 °C, ~ 430 °C)	Similar degradation trend, slight Tmax shift	Slightly enhanced Tmax and charring ability due to aromatic units	EKL-C1 improves thermal stability and fire resistance, enhancing durability for practical applications
FTIR (Hydroxyl Stretching Shift)	3298 cm⁻¹	Blue shift to 3314 cm⁻¹ at 15% LNP	Blue shift to 3316 cm⁻¹ at 15% LNP	Strong hydrogen bonding between LNPs and PVA, slightly more pronounced with EKL-C1, indicating better integration

Overall, the comparative evaluation demonstrates the superior performance of EKL-C1-based foils in terms of UV protection, nanoparticle dispersion, and structural integrity. These results confirm the potential of acetylated LNPs, particularly those derived from eucalyptus, as effective functional additives for sustainable, biodegradable packaging.

## Conclusions

This study demonstrates the successful development of UV-protective, transparent, and biodegradable PVA foils using acetylated lignin nanoparticles (LNPs) derived from spruce and eucalyptus. The incorporation of LNPs significantly enhanced the UV-shielding performance of PVA, with eucalyptus-derived EKL-C1 nanoparticles showing superior results due to their higher methoxyl content and more uniform dispersion in the polymer matrix.

Comprehensive characterization, including UV-Vis spectroscopy, AFM, TEM, and FTIR, confirmed that both lignin source and nanoparticle distribution play key roles in determining foil performance. EKL-C1-based films achieved stronger UV-blocking, smoother surfaces, and improved structural stability compared to SKL-C1. Mechanical and aging tests further underscored the effectiveness of LNPs in enhancing durability.

These findings highlight the potential of lignin nanoparticles, particularly from hardwood sources, as sustainable, high-performance additives for advanced packaging materials. The improved mechanical stability after UV exposure makes these composites promising candidates for protecting light-sensitive products, while also supporting lignin valorization and circular material strategies. Although barrier properties were not directly measured in this study, previous research suggests that well-dispersed lignin nanoparticles may also contribute to enhanced barrier performance in similar polymer systems.

Future research could focus on scaling up production, evaluating long-term UV stability, and exploring other applications for lignin-based nanocomposites. Tailoring lignin modification strategies and assessing alternative lignin sources may further enhance material performance and extend their potential in sustainable packaging and related fields.

## Supplementary Information

Below is the link to the electronic supplementary material.

Supplementary Material 1

## Data Availability

The datasets used and/or analysed during the current study are available from the corresponding author on reasonable request.
