# Supplementary material for: Lignin nanoparticle-enhanced PVA foils for UVB/UVC protection
Source: Sci Rep. 2025 Oct 13;15:35735. doi: 10.1038/s41598-025-19753-6 (PMC12518772; doi:10.1038/s41598-025-19753-6)
Supplement: Supplementary file 1 — Supplementary Material 1 [file 41598_2025_19753_MOESM1_ESM.docx]

**SUPPLEMENTARY MATERIALS**

Marta Goliszek-Chabros^1*^, Nataliia Smyk^2^, Taroan Xu^3^, Arkadiusz Matwijczuk^4,5^, Beata Podkościelna^6^, Olena Sevastyanova^3,7*^

***Lignin nanoparticle-enhanced PVA foils for UVB/UVC shielding***

*^1^Analytical Laboratory, Institute of Chemical Science, Faculty of Chemistry, Maria Curie-Skłodowska University, M. Curie-Skłodowska Sq. 3, 20-031 Lublin, Poland, e-mail: marta.goliszek-chabros@mail.umcs.pl;*

*^2^Department of Analytical Chemistry, Faculty of Chemistry, Taras Shevchenko National University of Kyiv, Hetman Pavlo Skoropadsky 12, 01033 Kyiv, Ukraine, e-mail:* *nataliiasmyk@knu.ua;*

*^3^Wallenberg Wood Science Center, Department of Fiber and Polymer Technology, School of Chemistry, Biotechnology and Health, KTH Royal Institute of Technology, Teknikringen 56-58, 100 44 Stockholm, Sweden, e-mail: olena@kth.se;*

*^4^Department of Biophysics, Institute of Molecular Biophysics, Faculty of Environmental Biology, University of Life Sciences in Lublin, Akademicka 13, 20-950 Lublin, Poland, e-mail:* *arkadiusz.matwijczuk@up.lublin.pl;*

*^5^ECOTECH-COMPLEX – Analytical and Program Center for Advanced Environmentally-Friendly Technologies, Maria Curie-Skłodowska University, Głęboka 39, 20-033 Lublin, Poland, e-mail:* *arkadiusz.matwijczuk@up.lublin.pl;*

*^6^Department of Polymer Chemistry, Institute of Chemical Science, Faculty of Chemistry, Maria Curie-Skłodowska University, M. Curie-Skłodowska Sq. 5, 20-031 Lublin, Poland, e-mail: beata.podkoscielna@mail.umcs.pl;*

*^7^Division of Wood Chemistry and Pulp Technology, Department of Fiber and Polymer Technology, School of Chemistry, Biotechnology and Health, KTH Royal Institute of Technology, Teknikringen 56-58, 100 44 Stockholm, Sweden, e-mail: olena@kth.se.*

**Correspondence o: marta.goliszek-chabros@mail.umcs.pl, olena@kth.se*

nLNP

**Figure S1**. UV light region transmittance rate at 365 nm and visible light region transmittance rate at 550 nm.

2SKL-C1

5SKL-C1

10SKL-C1

15SKL-C1

2EKL-C1

5EKL-C1

10EKL-C1

15EKL-C1

a b

**Figure S2**. Transparency spectra of PVA foils with varying concentrations of LNP

1. SKL-C1, b) EKL-C1.

**Table S1.** Color of LNP-PVA foils.

|  | nLNP | | | | |
| --- | --- | --- | --- | --- | --- |
|  | 0 | 2 | 5 | 10 | 15 |
| SKL-C1 Series | 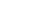 | 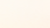 | 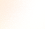 | 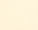 | 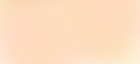 |
| EKL-C1 Series |  | 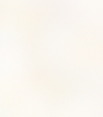 | 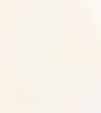 | 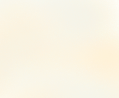 | 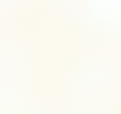 |

**Table S2.** Mechanical properties of the foils with SKL-C1 and with EKL-C1 nanoparticles before and after UV exposure.

| Sample | Condition | Tensile strength  (MPa) | Young’s modulus  (MPa) | Elongation at break  (%) |  |
| --- | --- | --- | --- | --- | --- |
| PVA (Ref) | Pre-UV | 28.19±1.44 | 1279±44 | 115.02±0.55 | |
|  | Post-UV | 16.2 ± 1.1 | 1201 ± 65 | 52.0 ± 3.2 | |
| 2SKL-C1 | Pre-UV | 28.59±0.28 | 1545±40 | 99.47±0.01 | |
|  | Post-UV | 22.4 ± 5.6 | 1312 ± 46 | 77.3 ± 0.9 | |
| 5SKL-C1 | Pre-UV | 27.52±1.04 | 1012±28 | 104.48±0.12 | |
|  | Post-UV | 19.0 ± 0.7 | 843 ± 17 | 74.5 ± 0.2 | |
| 10SKL-C1 | Pre-UV | 17.47±1.35 | 1457±169 | 100.49±0.06 | |
|  | Post-UV | 11.5 ± 1.3 | 1204 ± 17 | 72.6 ± 0.3 | |
| 15SKL-C1 | Pre-UV | 12.22±1.56 | 1245±47 | 95.29±0.02 | |
|  | Post-UV | 9.2 ± 1.3 | 1150 ± 11 | 72.4 ± 0.5 | |
| 2EKL-C1 | Pre-UV | 29.27±0.52 | 1160±44 | 92.68±0.10 | |
|  | Post-UV | 24.5 ± 6.2 | 1051 ± 43 | 77.5 ± 0.5 | |
| 5EKL-C1 | Pre-UV | 28.33±6.76 | 2103±227 | 100.79±0.29 | |
|  | Post-UV | 23.5 ± 1.4 | 1905 ± 32 | 82.5 ± 0.2 | |
| 10EKL-C1 | Pre-UV | 16.70±1.29 | 936±125 | 120.57±0.20 | |
|  | Post-UV | 13.7 ± 0.2 | 851 ± 23 | 93.5 ± 0.4 | |
| 15EKL-C1 | Pre-UV | 14.80±3.79 | 2102±356 | 99.45±0.12 | |
|  | Post-UV | 12.3 ± 3.1 | 1909 ± 73 | 81.3 ± 0.4 | |
